# A novel prognostic signature of cuproptosis-related genes and the prognostic value of FDX1 in gliomas

**DOI:** 10.3389/fgene.2022.992995

**Published:** 2022-12-12

**Authors:** HuaXin Zhu, Qinsi Wan, Jiacong Tan, Hengyang Ouyang, Xinyi Pan, MeiHua Li, YeYu Zhao

**Affiliations:** ^1^ Department of Neurosurgery, The First Affiliated Hospital of Nanchang University, Nanchang, Jiangxi, China; ^2^ Medical Innovation Center, The First Affiliated Hospital of Nanchang University, Nanchang, Jiangxi, China; ^3^ Department of Gastroenterology, The First Affiliated Hospital of Nanchang University, Nanchang, Jiangxi, China; ^4^ Huankui Academy, Nanchang University, Nanchang, Jiangxi, China

**Keywords:** gliomas, cuproptosis, FDX1, prognosis, prognostic signature

## Abstract

**Background:** Gliomas are the most common malignant tumors of the central nervous system, with extremely bad prognoses. Cuproptosis is a novel form of regulated cell death. The impact of cuproptosis-related genes on glioma development has not been reported.

**Methods:** The TCGA, GTEx, and CGGA databases were used to retrieve transcriptomic expression data. We employed Cox’s regressions to determine the associations between clinical factors and cuproptosis-related gene expression. Overall survival (OS), disease-specific survival (DSS), and progression-free interval (PFI) were evaluated using the Kaplan-Meier method. We also used the least absolute shrinkage and selection operator (LASSO) regression technique.

**Results:** The expression levels of all 10 CRGs varied considerably between glioma tumors and healthy tissues. In glioma patients, the levels of CDKN2A, FDX1, DLD, DLAT, LIAS, LIPT1, and PDHA1 were significantly associated with the OS, disease-specific survival, and progression-free interval. We used LASSO Cox’s regression to create a prognostic model; the risk score was (0.882340) *FDX1 expression + (0.141089) *DLD expression + (–0.333875) *LIAS expression + (0.356469) *LIPT1 expression + (–0.123851) *PDHA1 expression. A high-risk score/signature was associated with poor OS (hazard ratio = 3.50, 95% confidence interval 2, –4.55, log-rank *p* < 0.001). Cox’s regression revealed that the FDX1 level independently predicted prognosis; FDX1 may control immune cell infiltration of the tumor microenvironment.

**Conclusion:** The CRG signature may be prognostic in glioma patients, and the FDX1 level may independently predict glioma prognosis. These data may afford new insights into treatment.

## 1 Introduction

Malignant tumors of the central nervous system (CNS) have one of the poorest prognoses of all cancers; life is shortened by about 20 years (Rouse et al., 2010; Reifenberger et al., 2017). More than 70% of all malignant CNS tumors are gliomas; such tumors are the most common CNS tumors (Gilbert et al., 2014). Half of all newly diagnosed gliomas are very malignant glioblastomas; the median patient survival is approximately 12 months (Gramatzki et al., 2016; Quinones and Le, 2018). Over the last decade, isocitrate dehydrogenase (IDH) mutations, chromosome 1p/19q deletions, MGMT and TERT promoter methylations, and histone mutations have all served as glioma biomarkers; these markers play key roles in glioma classification and treatment decisions (Chen et al., 2017; Brito et al., 2019). Despite great advances in surgery, radiotherapy, chemotherapy, and targeted therapy, almost all malignant gliomas recur, associated with poor prognoses. Better prognostic models are urgently required.

Although heavy metal ions are crucial micronutrients, ion levels that are too low or excessive may trigger controlled cell death (Wang et al., 2022a) *via* activation of various subprograms. For example, ferroptosis is an iron-dependent form of uncontrolled, lipid peroxidation-induced, oxidative cell death (Dixon et al., 2012; Liang et al., 2019). Recently, Tsvetkov et al. (2022) found that intracellular copper (Cu) triggered a unique form of controlled cell death termed “cuproptosis,” which differed from apoptosis, necrosis, autophagy, and ferroptosis. The lipoylated acylated components of the tricarboxylic acid (TCA) cycle bind directly to copper, imparting protein stress and eventual cell death (Hatori et al., 2016). Recent studies have shown that cancer patients exhibit much higher serum and tumor tissue copper levels than healthy people (Blockhuys et al., 2017; Ge et al., 2022). Dysregulation of copper homeostasis may be cytotoxic; changes in intracellular copper levels may influence cancer development and spread (Ishida et al., 2013; Babak and Ahn, 2021). However, any relationship between cuproptosis and glioma progression remains unknown. This is our topic here; glioma cuproptosis-related gene (CRG) expression is of clinical and potential prognostic utility.

## 2 Methods

### 2.1 Data acquisition

The glioma RNA-seq data of TCGA and the corresponding normal tissue data of GTEx derived *via* uniform toil processing were downloaded from UCSCXENA (https://xenabrowser.net/datapages/). The Human Protein Atlas database (https://www.proteinatlas.org/) was used to examine protein expression in normal and glioma tissues. We also downloaded RNA-seq data and clinical information (DataSet ID: mRNAseq_693) of glioma samples from the CGGA database (http://www.cgga.org.cn/); we used this information to externally validate the survival analyses.

### 2.2 Differential expression analysis

We integrated the TCGA and GTEx databases and then sought differences in CRG expression levels between glioma and normal tissue samples. We drew receiver operating characteristic (ROC) curves to assess the predictive accuracies of CRG levels. The relationships between CRG levels and clinicopathological features were explored using the TCGA database and validated employing the CGGA database. The R package DESeq2 (ver. 1.26.0) was used to distinguish the expression levels of mRNAs associated with low and high risks of progression; we identified differentially expressed genes (DEGs) using the thresholds of log_2_ (FC) > 2.0 and an adjusted *p*-value <0.05. The DEGs were displayed using volcano plots and heat maps.

### 2.3 Prognostic signatures of cuproptosis-related genes

Useful features were selected using the least absolute shrinkage and selection operator (LASSO) regression algorithm; 10-fold cross-validation followed and the R package glmnet was used to analyze the data. First, multifactorial Cox’s regression was performed, followed by iteration using a step function. The optimal model was selected. We used the log-rank test and Cox’s proportional hazards regression to derive *p*-values and hazard ratios with 95% confidence intervals (CIs); then we drew Kaplan-Meier curves. We used the decision curve analysis R package ggDCA to construct the prognostic signature of CRG levels.

### 2.4 Functional and pathway enrichment analyses

We employed the R clusterProfiler package to perform Gene Ontology (GO) and Kyoto Encyclopedia of Genes and Genomes (KEGG) enrichment analyses and displayed the findings using the R packages dplyr and ggplot2. We uploaded the DEGs to Metascape (an online tool for gene function/annotation analysis; https://metascape.org/).

### 2.5 Construction of a prognostic model and external validation

We sought prognostic factors among clinical variables, performed univariate and multivariate Cox’s regressions, and derived an optimal prognostic model. The R package rms was used to create a nomogram that predicted prognosis. Harrell concordance index (C-index) calibration plots were drawn to evaluate the reliabilities and accuracies of the prognostic models. Kaplan-Meier curves were drawn to compare the differences between paired groups in terms of overall survival (OS), disease-specific survival (DSS), and progression-free interval (PFI).

### 2.6 Evaluation of immune cell infiltration

The Tumor Immune Estimation Resource (Timer) method of the R package immunedeconv was used to seek correlations between immune cell activities, and the constructed models and the gene expression levels *per se*. The R package ggstatsplot was employed to derive correlations between gene expression levels and immune system scores; the R package pheatmap was used to identify multi-gene correlations. We used the single-sample Gene Set Enrichment Analysis (ssGSEA) technique of the R GSVA package to measure the levels of 24 immune cell types associated with glioma infiltration. The R package is based on the TCGA database. We used Spearman analyses to derive correlations between quantitative variables that were not normally distributed.

### 2.7 Statistical analysis

Paired samples were evaluated using the Wilcoxon signed-rank test; unpaired samples were assessed employing the Wilcoxon rank-sum test. We used the Kruskal–Wallis test, the Wilcoxon signed-rank test, and logistic regression to explore the relationships between clinical characteristics and CRG expression levels. Either the chi-square or the Fisher exact test was used to investigate relationships between CRG expression levels and the clinical characteristics. R software ver. 4.0.3 was employed for all statistical analyses (R Foundation for Statistical Computing, Vienna, Austria). A *p*-value <0.05 was considered significant.

## 3 Results

### 3.1 CRG expression levels in glioma patients

**FIGURE 1:**
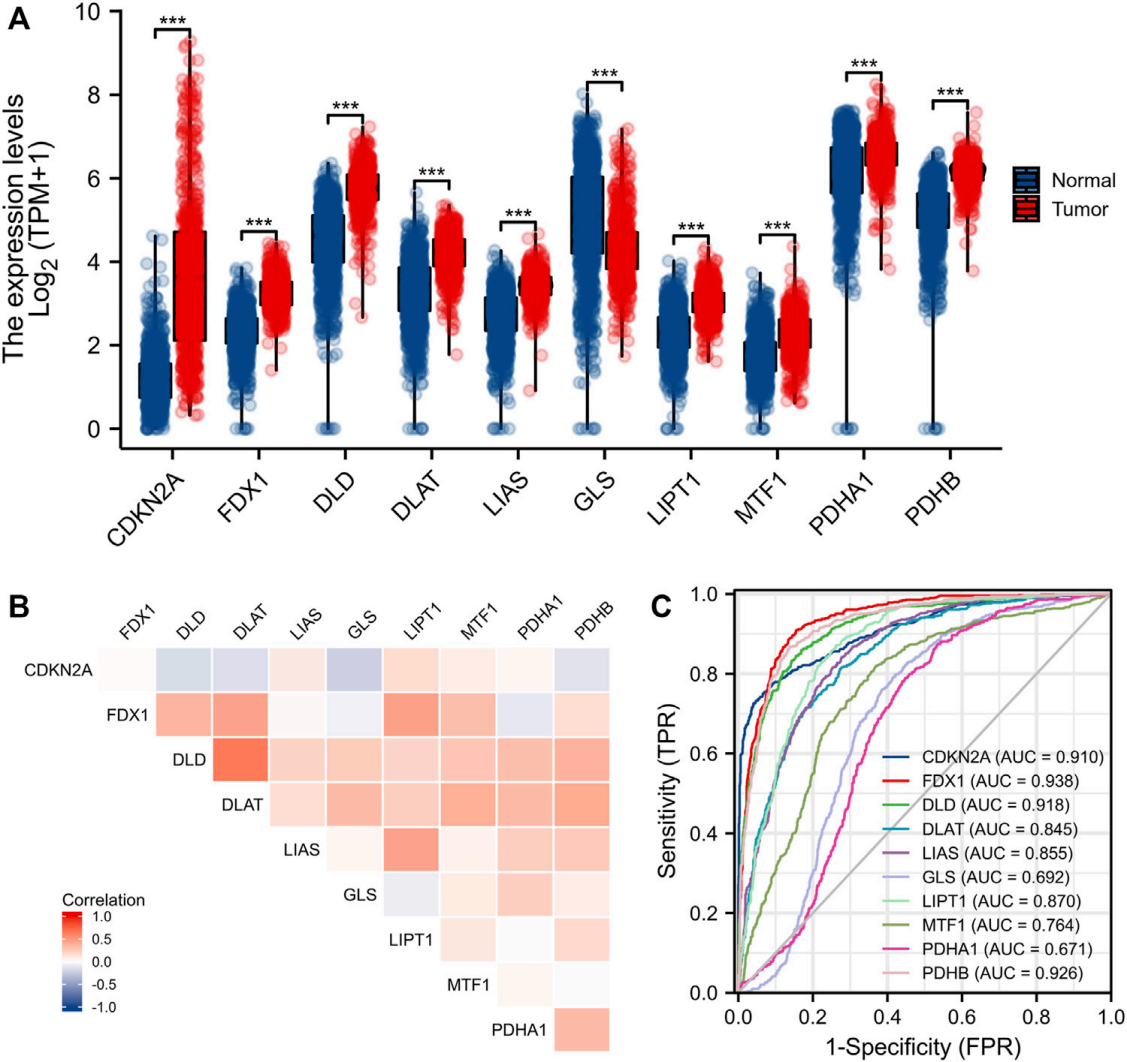
Expression of CRGs in gliomas. **(A)** Expression of 10 CRGs in gliomas and normal tissues. **(B)** Correlation between CRGs expression. **(C)** Diagnostic value of CRGs in gliomas. *p < 0.05, **p < 0.01, ***p < 0.001. CRGs: cuproptosis-related genes.

**FIGURE 2:**
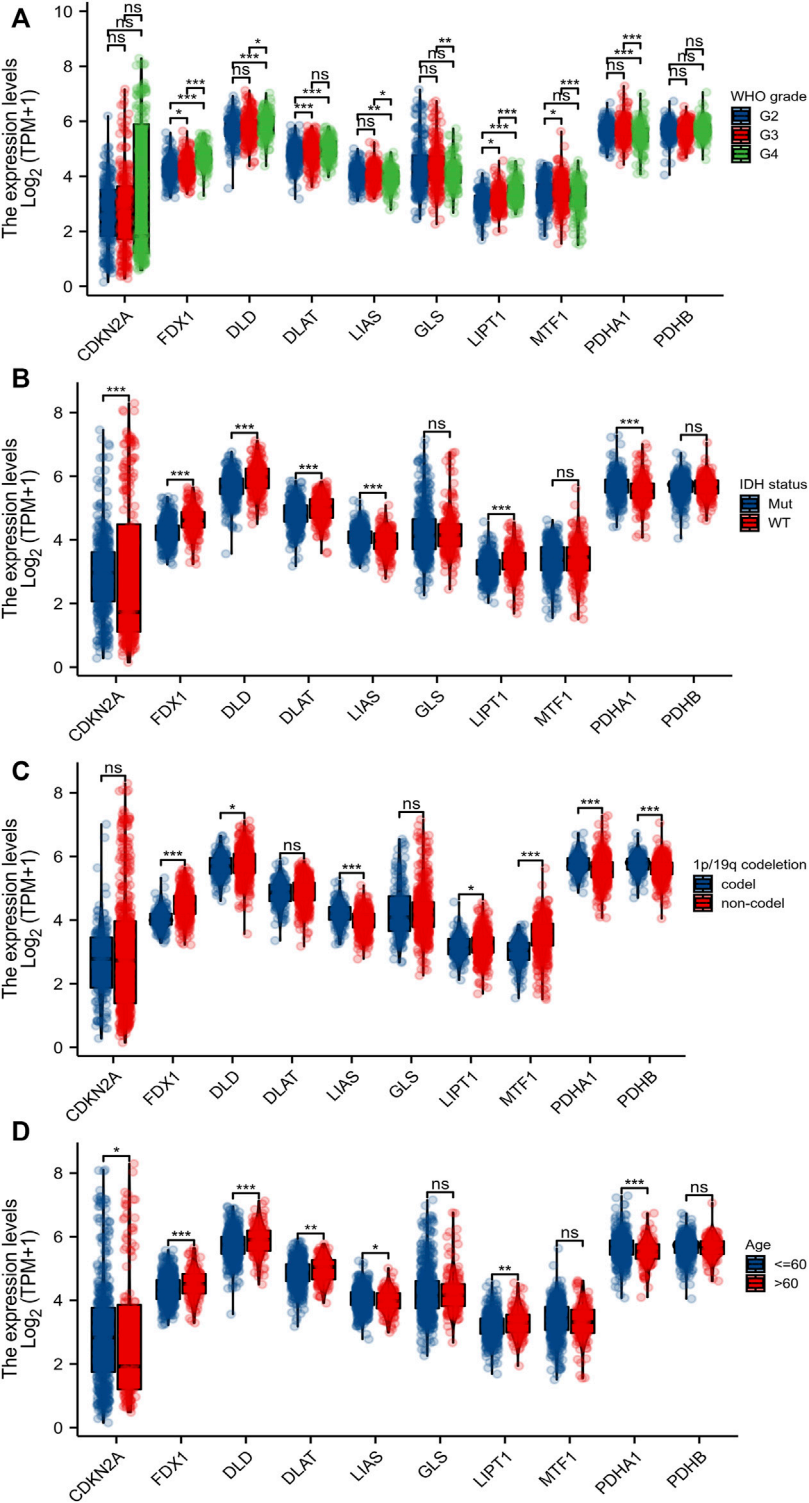
Relationship between CRGs and clinicopathological features. The association of 10 CRGs expression with WHO grade **(A)**, IDH status **(B)**, 1p/19q codeletion **(C)**, and age **(D)** in gliomas from TCGA database. *p < 0.05, **p < 0.01, ***p < 0.001. CRGs: cuproptosis-related genes.

Eleven genes (CDKN2A, DLD, DLAT, FDX1, GLS, LIAS, LIPT1, MTF1, PDHA1, and PDHB) are closely associated with cuproptosis. We found that only the expression level of GLS was significantly decreased in gliomas (compared to normal tissues); the levels of all other CRGs increased significantly (Figure 1A). We sought correlations between the levels of different CRGs (Figure 1B). We performed receiver operating characteristic curve (ROC) analyses to assess whether various CRG levels aided glioma detection; the areas under the curves (AUCs) were >0.9 for CDKN2A, FDX1, DLD, and PDHB (Figure 1C). Levels of FDX1, LIAS, LIPT1, DLD, DLAT, MTF1, and PDHA1 were associated with the World Health Organization (WHO) clinical grade (Figure 2A); levels of CDKN2A, DLAT, LIAS, LIPT1 FDX1, DLD, and PDHA1 with were associated with IDH status (Figure 2B); levels of FDX1, LIPT1, MTF1, PDHA1, DLD, LIAS, and PDHB were associated with 1p19q co-deletion (Figure 2C); and levels of CDKN2A, DLD, DLAT, LIAS, LIPT1, FDX1 and PDHA1 levels were associated with age (Figure 2D).

### 3.2 The survival analyses of CRGs in gliomas patients

**FIGURE 3:**
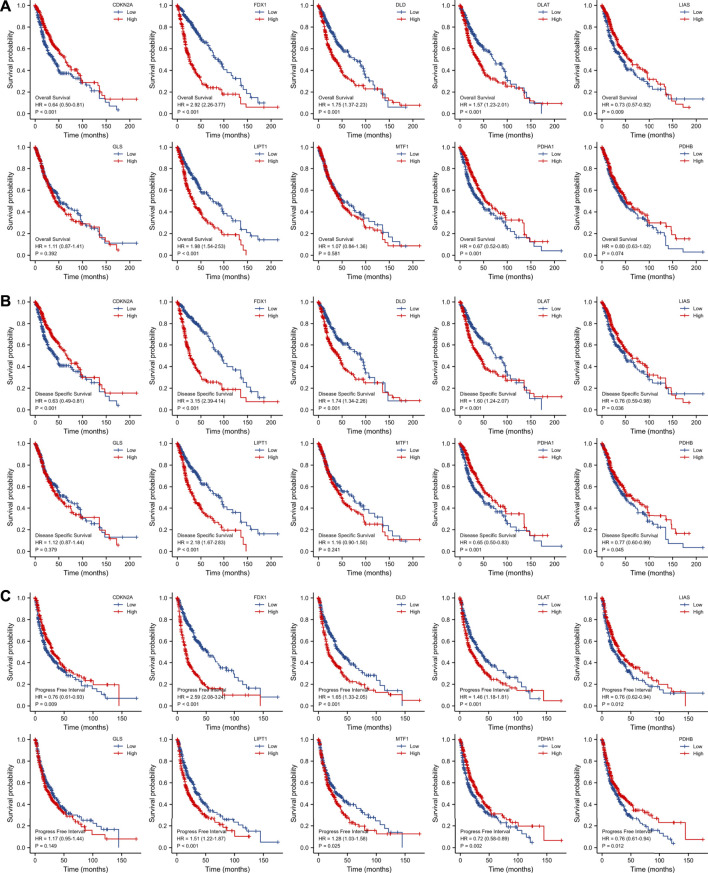
Survival analysis of OS **(A)**, DSS **(B)** and PFI **(C)** of 10 CRGs in gliomas patients from TCGA database. OS: overall survival, DSS: disease specific survival, PFI: progress free interval, CRGs: cuproptosis-related genes.

Patients of the TCGA database expressing high levels of FDX1 (hazard ratio [HR] = 2.92 [95% confidence interval, CI] 2.26–3.77, log-rank *p* < 0.001); DLD (HR = 1.75 [1.37–2.23], log-rank *p* < 0.001); DLAT (HR = 1.57 [1.23–2.01], log-rank *p* < 0.001); and LIPT1 [HR = 1.98 (1.54–2.53), log-rank *p* < 0.001]; and low levels of CDKN2A [HR = 0.64 (0.50–0.81), log-rank *p* < 0.001]; LIAS [HR = 0.73 (0.57–0.92), log-rank *p* = 0.009]; and PDHA1 [HR = 0.67 (0.52–0.85), log-rank *p* = 0.001] experienced poorer OS than others (Figure 3A). Poor DSS was associated with high-level expression of FDX1 [HR = 3.15 (2.39–4.14), log-rank *p* < 0.001]; DLD [HR = 1.74 (1.34–2.26), log-rank *p* < 0.001]; DLAT [HR = 1.60 (1.24–2.07), log-rank *p* < 0.001]; and LIPT1 [HR = 2.18 (1.67–2.83), log-rank *p* < 0.001]; and low-level expression of CDKN2A [HR = 0.63 (0.49–0.81), log-rank *p* < 0.001]; LIAS [HR = 0.76 (0.59–0.98), log-rank *p* = 0.036]; PDHA1 [HR = 0.65 (0.50–0.83), log-rank *p* = 0.001]; and PDHB [HR = 0.77 (0.60–0.99), log-rank *p* = 0.045] (Figure 3B). PFI was affected by the levels of CDKN2A [HR = 0.76 (0.61–0.93), log-rank *p* = 0.009]; FDX1 [HR = 2.59 (2.08–3.24), log-rank *p* < 0.001]; DLD [HR = 1.65 (1.33–2.05), log-rank *p* < 0.001]; DLAT [HR = 1.46 (1.18–1.81), log-rank *p* < 0.001]; LIAS [HR = 0.76 (0.62–0.94), log-rank *p* = 0.012]; LIPT1 [HR = 0.51 (1.22–1.87), log-rank *p* < 0.001]; MTF1 [HR = 1.28 (1.03–1.58), log-rank *p* < 0.001]; PDHA1 [HR = 0.72 (0.58–0.89), log-rank *p* = 0.002]; and PDHB [HR = 0.76 (0.61–0.94), log-rank *p* = 0.012] (Figure 3C).

### 3.3 Construction of a CRG-derived prognostic gene signature

**FIGURE 4:**
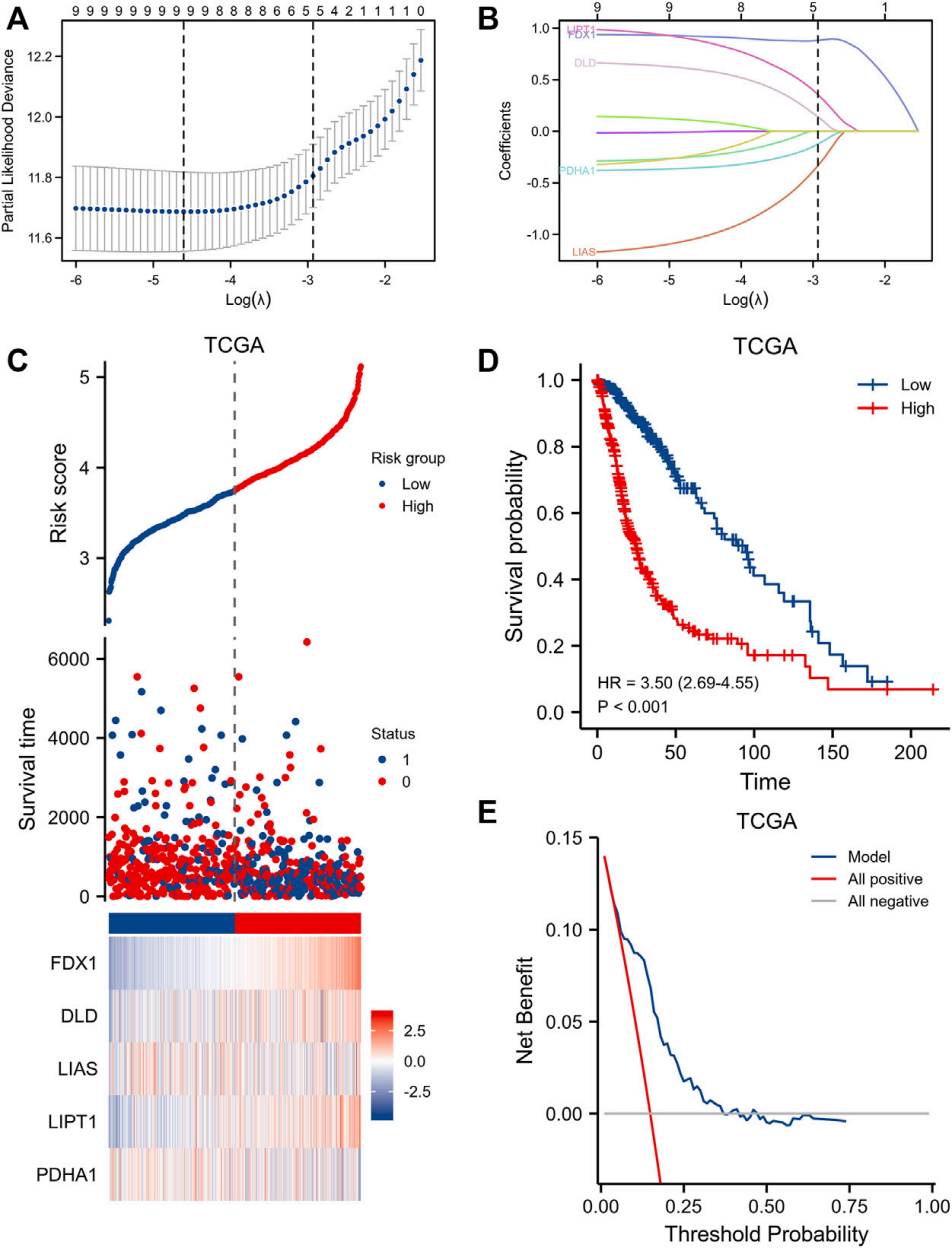
Clinical relevance of CRGs in the gliomas patients of TCGA. **(A,B)** LASSO regression analysis and partial likelihood deviance of the CRGs. **(C)** distribution of risk score, survival status and the expression of prognostic gliomas, **(D)** Kaplan−Meier plot of the CRGs signature and overall survival, **(E)** Decision curve analysis of CRGs signature for predicting survival status.

LASSO Cox’s regression generated a five-gene signature (Figures 4A, B): Risk Score = (0.882340) *FDX1 expression + (0.141089) *DLD expression + (–0.333875) *LIAS expression + (0.356469) *LIPT1 expression + (–0.123851) *PDHA1 expression. We found a significant correlation between a high risk score and poor OS [HR = 3.50 (2.69–4.55), log-rank *p* < 0.001] (Figures 4C,D). Decision curve analysis in terms of OS prediction showed that the signature performed well [C-index 0.758 (0.743–0.772)] (Figure 4E). The CGGA database was utilized for external validation; we retrieved all data on glioma patients. As for TCGA patients, CGGA individuals with higher risk scores evidenced significantly shorter OS [HR = 1.68 (1.38–2.05), log-rank *p* < 0.001] (Supplementary Figure S1). The clinical information of TCGA and CGGA datasets were provided in Supplementary Tables S1, S2.

### 3.4 Functional enrichment and immune cell infiltration associated with high and low signature risk scores

**FIGURE 5:**
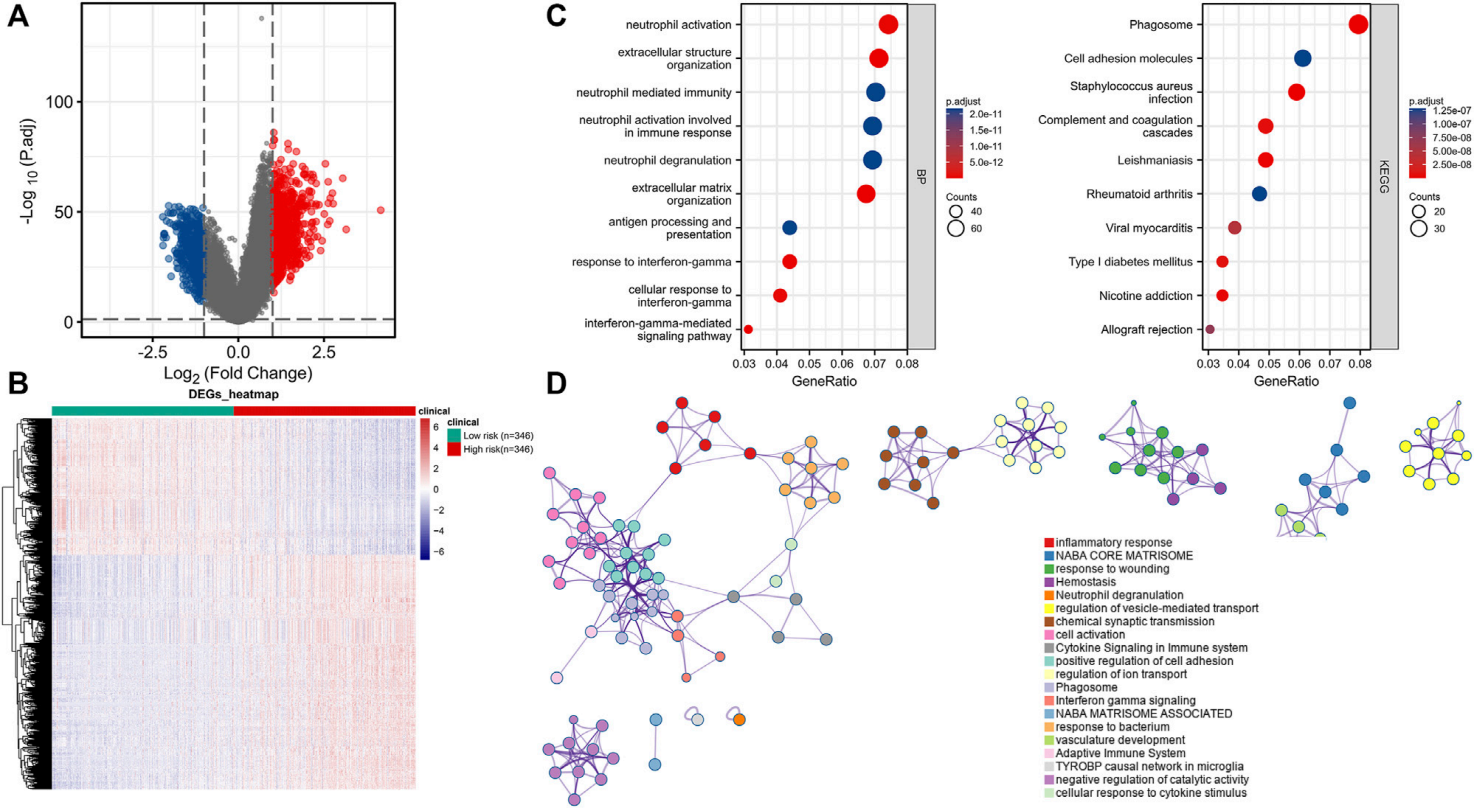
Functional enrichment analysis for CRGs signature in gliomas patients. **(A)** Volcano Plot of differentially expressed genes (DEGs) screened based on CRGs signature. **(B)** Gene set enrichment analysis of Gene Ontology biological process (GO-BP). **(C)** Gene set enrichment analysis of Kyoto Encyclopedia of Genes and Genomes (KEGG). **(D)** Network and bar chart of enrichment analysis results drawn by Metascape.

**FIGURE 6:**
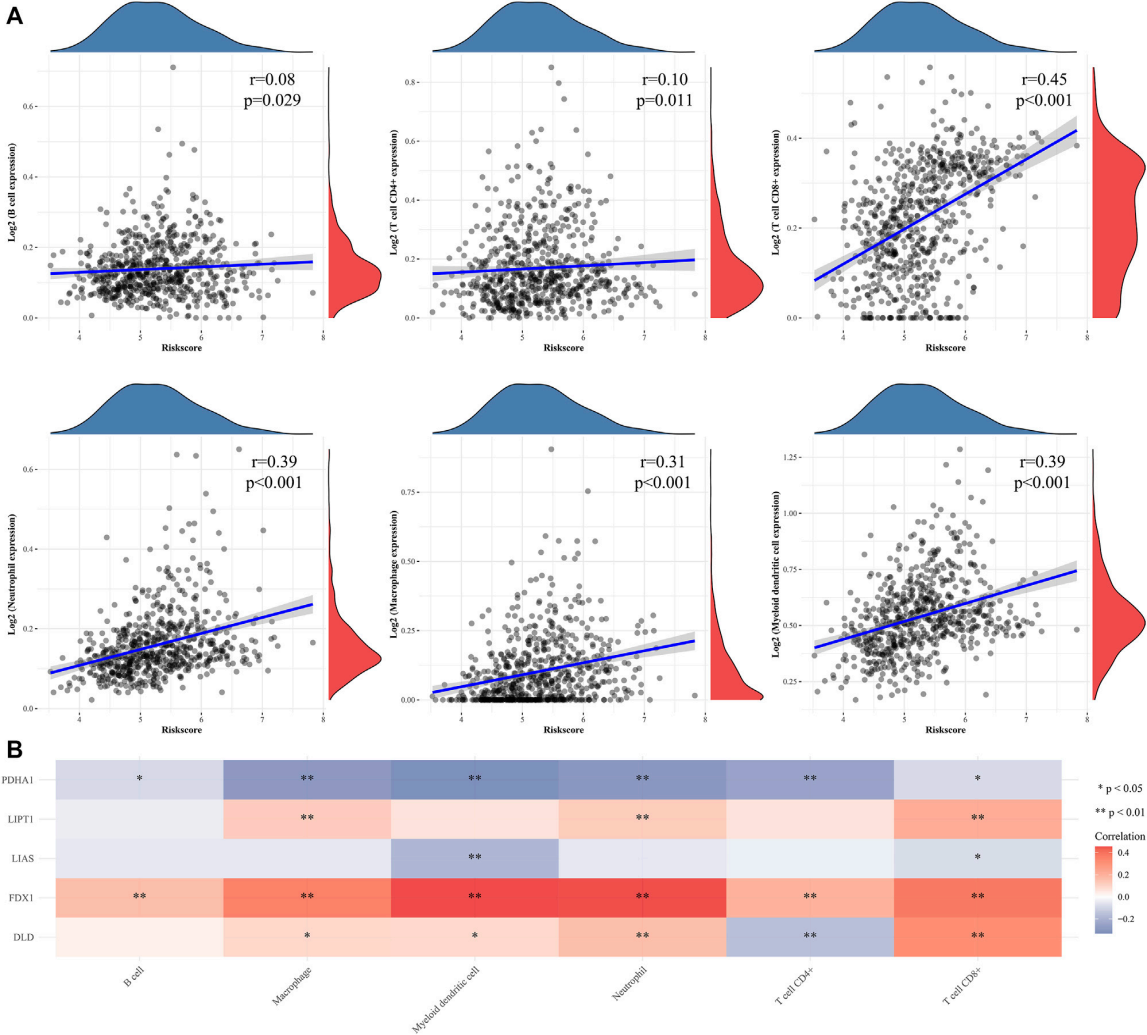
Immune infiltration analyse of CRGs signature in gliomas patients. **(A)** The correlations between CRGs signature risk score and immune cell was analysed with Spearman. **(B)** A heatmap of the correlation between CRGs signature genes and immune score. *p < 0.05, **p < 0.01, ***p < 0.001.

We measured the differentially expressed (DE) mRNA levels of gliomas with high and low CRG signature risk scores to explore whether cuproptosis might trigger glioma progression. A volcano plot (Figure 5A) and a heat-map (Figure 5B) identified 1213 DE mRNAs, the expression of 767 of which were increased and 446 decreased (Supplementary Table S3). We sought gene enrichments in terms of biological processes (BP), cellular components (CC), molecular function (MF), and of the criteria of the KEGG (Supplementary Table S4). Neutrophil activation, extracellular structure organization, neutrophil mediated immunity, neutrophil activation involved in immune response, neutrophil degranulation and extracellular matrix organization were all significantly activated on GO-BP enrichment analysis. Phagosome, cell adhesion molecules, *staphylococcus aureus* infection, complement and coagulation cascades and leishmaniasis were enriched on KEGG. The results are shown in Figure 5C. Enrichment results drawn by Metascape were similar (Figure 5D). Immune cell infiltration analysis revealed that the CRG signatures were associated with all six immune cell types, but principally CD8^+^ T cells (Figure 6A). Figure 6B shows the correlations between the expression levels of CRG signature genes and immune cell activities.

### 3.5 The nomogram development and construction for gliomas patients

**FIGURE 7:**
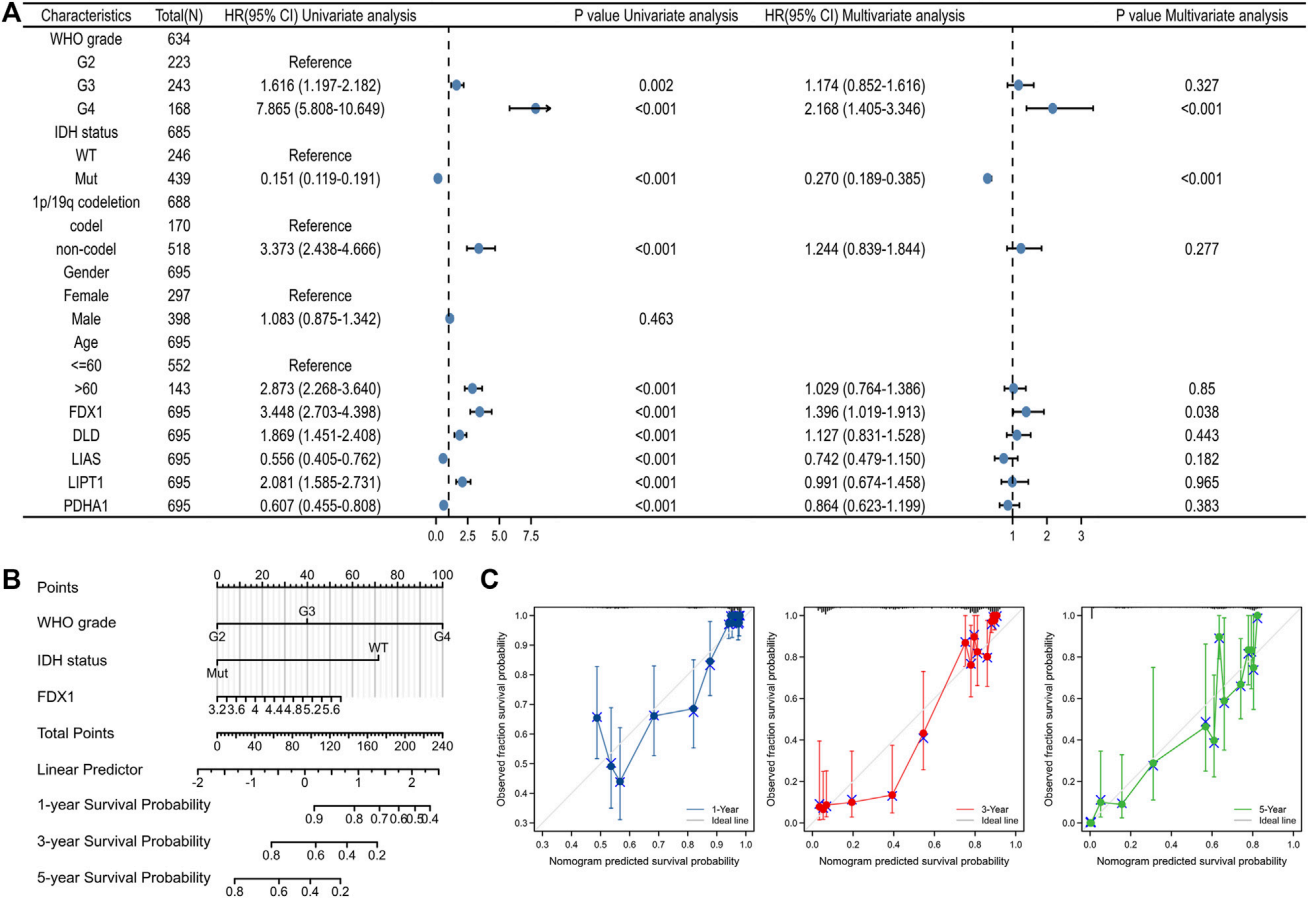
Construction of a predictive nomogram. **(A)** The univariate and multivariate Cox regression analysis for clinical parameters of gliomas and five CRGs. **(B)** Nomogram to predict the 1, 3, and 5 years overall survival rate of gliomas patients. **(C)** The calibration curve of the nomogram.

Univariate Cox’s regression revealed that a high WHO grade; wild-type IDH status; 1p19q non-co-deletion; age >60 years; high-level expression of FDX1, DLD, and LIPT1; and low-level expression of LIAS and PDHA1 were all associated with poor OS. Multivariate Cox’s HR analysis revealed that the WHO grade, IDH status, and the FDX1 mRNA expression level independently predicted OS (Figure 7A). We created an OS nomogram to integrate the FDX1 level with other prognostic factors (the WHO and IDH data) (Figure 7B). We drew a calibration curve to evaluate the contribution of the FDX1 level to nomogram performance; the C-index was 0.834 (0.823–0.846) (Figure 7C). The association between the FDX1 level and the clinicopathological parameters of TCGA patients with gliomas are shown in Table 1. Logistic regression analysis of the FDX1 data revealed strong associations with clinical characteristics including the WHO grade [OR = 3.634 (2.579–5.161), *p* < 0.001]; IDH status [OR = 5.945 (4.20–8.509), *p* < 0.001]; 1p/19q co-deletion [OR = 10.231 (6.492–16.801), *p* < 0.001]; and age (OR = 2.016 (1.384–2.961), *p* < 0.001) (Table 2). Thus, increased FDX1 expression was associated with poor prognosis.

**TABLE 1:** Association of FDX1 expression and clinicopathological parameters in patients with gliomas.

Characteristic	Low expression of FDX1	High expression of FDX1	*p*
*n*	348	348	
WHO grade, *n* (%)			<0.001
G2	155 (24.4%)	69 (10.9%)	
G3	130 (20.5%)	113 (17.8%)	
G4	27 (4.3%)	141 (22.2%)	
IDH status, *n* (%)			<0.001
WT	59 (8.6%)	187 (27.3%)	
Mut	287 (41.8%)	153 (22.3%)	
1p/19q codeletion, *n* (%)			<0.001
Codel	148 (21.5%)	23 (3.3%)	
non-codel	200 (29%)	318 (46.2%)	
Gender, *n* (%)			0.818
Female	147 (21.1%)	151 (21.7%)	
Male	201 (28.9%)	197 (28.3%)	
Age, *n* (%)			<0.001
≤60	296 (42.5%)	257 (36.9%)	
>60	52 (7.5%)	91 (13.1%)	
OS event, *n* (%)			<0.001
Alive	262 (37.6%)	162 (23.3%)	
Dead	86 (12.4%)	186 (26.7%)	
DSS event, *n* (%)			<0.001
Alive	266 (39.4%)	165 (24.4%)	
Dead	74 (11%)	170 (25.2%)	
PFI event, *n* (%)			<0.001
Alive	224 (32.2%)	126 (18.1%)	
Dead	124 (17.8%)	222 (31.9%)	
Age, median (IQR)	42 (33, 54)	50 (36, 62)	<0.001

**TABLE 2:** Logistic regression analysis of FDX1 expression.

Characteristics	Total (N)	Odds ratio (OR)	*p*-Value
WHO grade (G3&G4 vs. G2)	635	3.634 (2.579–5.161)	<0.001
IDH status (WT vs. Mut)	686	5.945 (4.20.-8.509)	<0.001
1p/19q codeletion (non-codel vs. codel)	689	10.231 (6.492–16.801)	<0.001
Age (>60 vs. ≤ 60)	696	2.016 (1.384–2.961)	<0.001
Gender (Male vs. Female)	696	0.954 (0.706–1.288)	0.759

### 3.6 Validation of the effects of FDX1 levels as revealed by the CGGA and HPA databases

**FIGURE 8:**
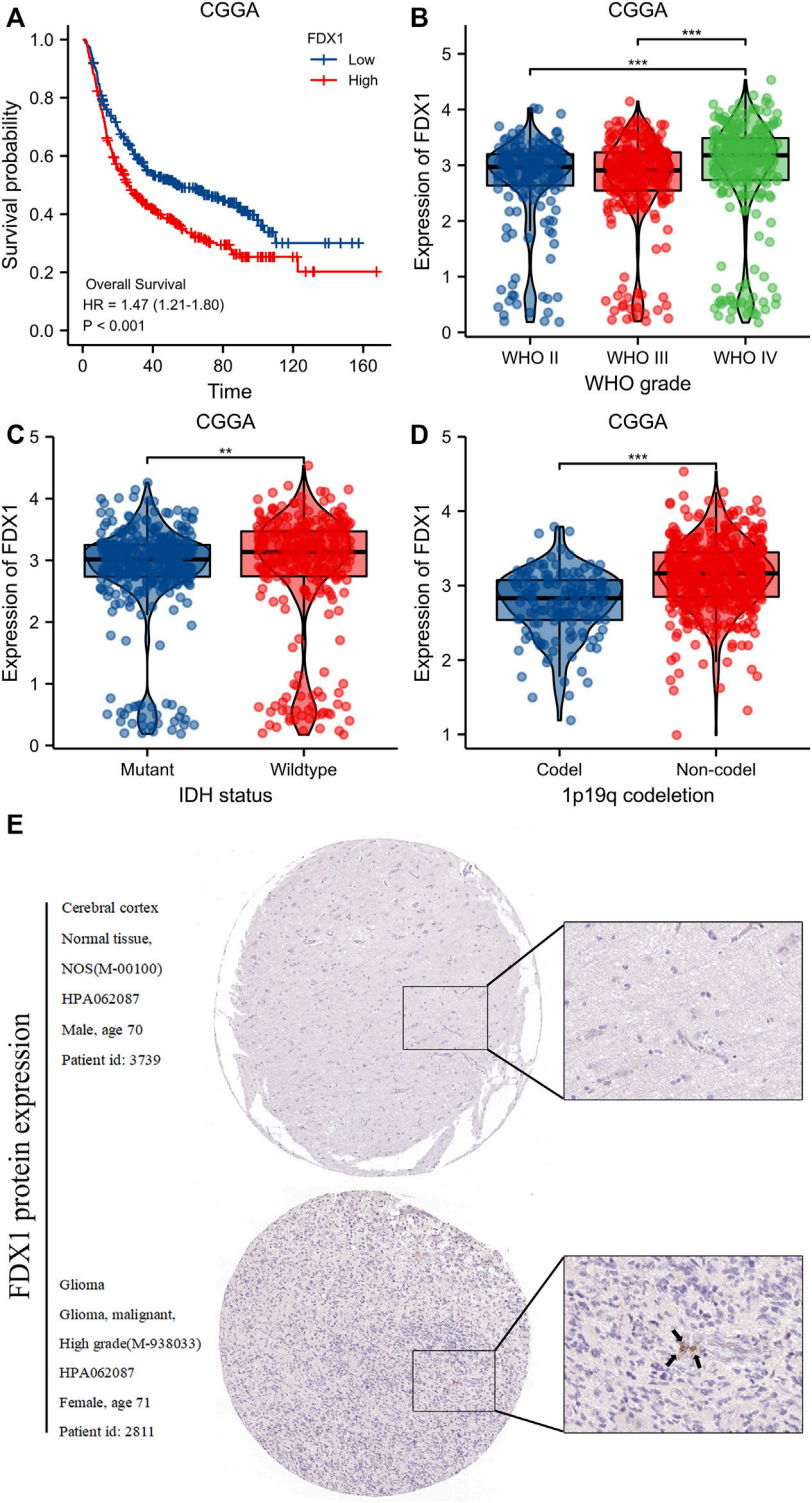
Validation of FDX1 expression from CGGA and HPA database. **(A)**Kaplan-Meier survival curve analysis of overall survival (OS) showed that high FDX1 expression correlated to poor prognosis of gliomas patients from CGGA database. **(B–D)** The association of FDX1 expression with WHO grade **(B)**, IDH status **(C)** and 1p/19q codeletion **(D)** in gliomas from CGGA database. **(E)** FDX1 protein expression in gliomas tissues determined using HPA. ***p < 0.001, ns, no statistical difference.

Compared to the low-level FDX1 group of the CGGA database, the high-level FDX1 group evidenced poorer OS [HR = 1.47 (1.12–1.80), log-rank *p* < 0.001) (Figure 8A). The FDX1 level was significantly correlated with the WHO grade (*p* < 0.001) (Figure 8B), IDH status (*p* < 0.01) (Figure 8C), and 1p19q co-deletion status (*p* < 0.001) (Figure 8D). Immunohistochemically, FDX1 staining was positive in gliomas but negative in normal tissues (Figure 8E).

### 3.7 Correlation between FDX1 expression levels and immune cell infiltration

**FIGURE 9:**
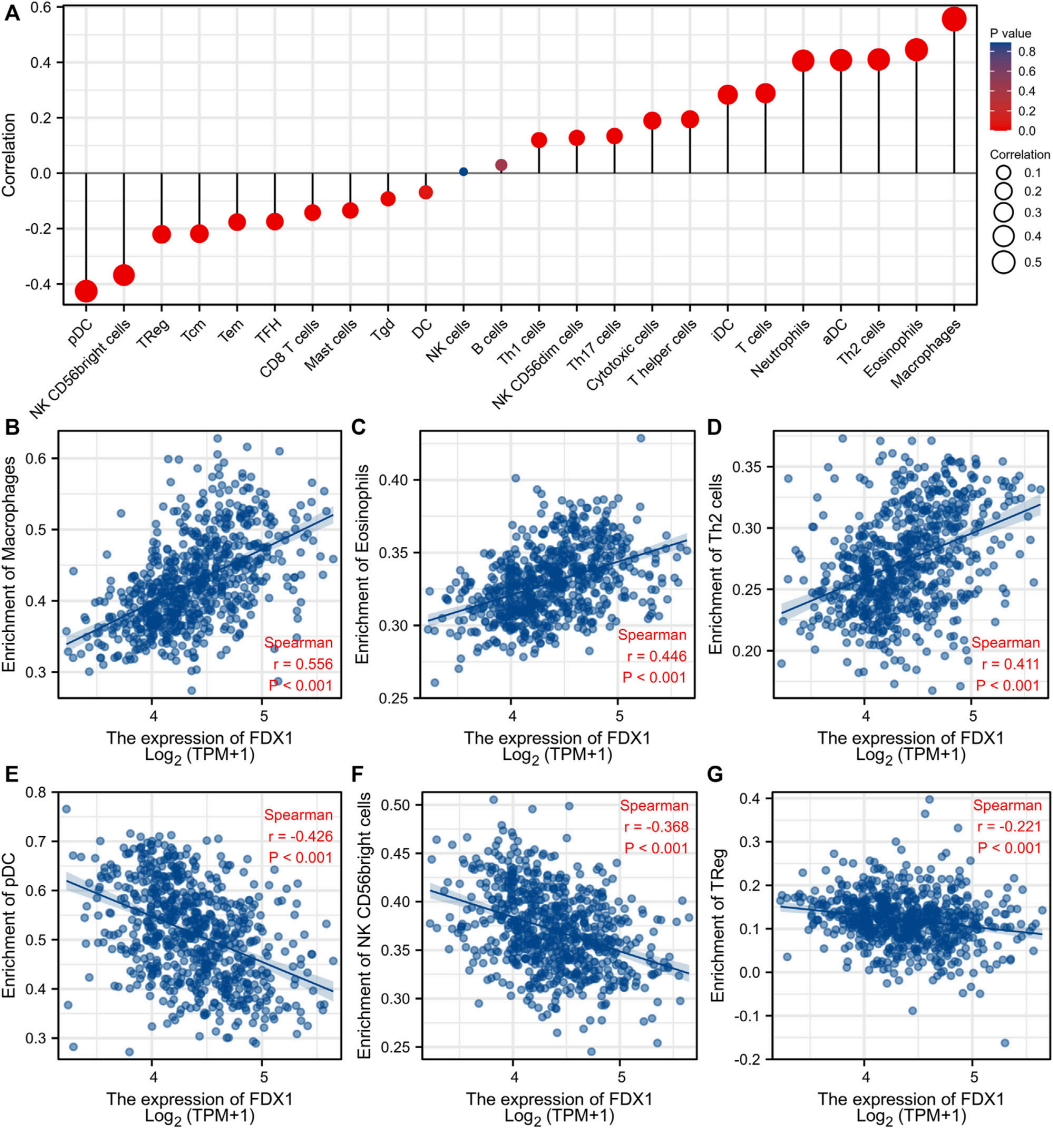
Association analysis of FDX1 expression and immune infiltration in gliomas patients. **(A)** The association between FDX1 expression and 24 tumor-infiltrating lymphocytes. **(B–D)** The positive correlation of FDX1 expression with immune infiltration level of Macrophages **(B)**, Eosinophils **(C)**, and Th2 cells**(D)**. **(E–G)** The negative correlation of FDX1 expression with immune infiltration level of pDC cells **(E)**, NK CD56 bright cells **(F)**, and TReg **(G)**.

The correlations between FDX1 expression levels and the numbers of immune cells of 24 different types were assessed. Figure 9A shows the results. The numbers of macrophages, eosinophils, and Th2 cells were significantly positively correlated with the FDX1 expression levels whereas the numbers of pDCs, NK CD56 bright cells, and Treg cells were significantly negatively correlated (Figure 9).

## 4 Discussion

Gliomas are the most common malignant tumors of the central nervous system, progress rapidly, and are associated with poor prognoses and high mortality, imposing significant costs on families and society (Lah et al., 2020; Guo et al., 2021). Early diagnosis, and timely and effective treatment, are critical. Improvements are essential (Muller et al., 2020). Copper binds to the acylated fatty acids of the TCA cycle, triggering toxic protein stress; this is cuproptosis (Tsvetkov et al., 2022). However, any role for cuproptosis in glioma progression remains poorly understood. We explored the glioma expression levels of 10 CRGs and their prognostic significances. We present a novel, cuproptosis-based, prognostic scoring system. We found that FDX1 might be a valuable therapeutic target.

The expression levels of all 10 CRGs differed between glioma and normal tissues. In addition to the levels of GLS, MTF1, and PDHB, those of the other seven CRGs significantly affected the OS, DSS, and PFI of glioma patients; cuproptosis may play a major role in disease progression. The signature risk scores showed that cuproptosis was highly correlated with tumor immune cell infiltration; cuproptosis may modulate such infiltration.

The “cuproptosis” concept is rather new; few studies on the effects of cuproptosis genes on glioma development have been published. Wang et al. (2022b) subjected CRG data to cluster analysis, and studied the possible roles of genomic mutations and immune cell infiltration in great depth; that study was very valuable. Chen et al. (2022) found that CRG levels were highly correlated with glioma aggressiveness and immune infiltration, and screened potential therapeutic agents, opening a new path toward glioma treatment. Ye et al. (2022) developed a valuable copper death prognostic model for WHO grade 2/3 glioma patients; drug-sensitivity was important. Ouyang et al. (2022) presented models based on lncRNA and CRG levels; cuproptosis-associated glutaminase gene expression reflected the prognosis of glioma patients. The prognostic model of Yan et al. (2022) used the levels of cuproptosis-associated lncRNAs to identify appropriate immune checkpoint blockade (ICB) therapies for LGG patients. We employed LASSO Cox’s regression analysis when constructing our prognostic model; the risk score was based on the levels of five CRGs (FDX1, DLD, LIAS, LIPT1, and PDHA1). Our model well-predicted the OS of glioma patients, not only those of the TCGA database but also those of the CGGA database. Our model yields risk scores (numbers), facilitating generalization. In addition, we found that FDX1 status was important in terms of glioma progression.

FDX1 encodes an iron-sulfur protein that reduces Cu^2+^ to Cu^1+^ and regulates protein acylation during cuproptosis (Dorsam and Fahrer, 2016; Weger et al., 2018). DLD is a key enzyme of many dehydrogenase and glycine decarboxylase complexes that maintain cellular and mitochondrial homeostasis (Duarte et al., 2021). LIPT1 and LIAS are members of the lipoic acid metabolic pathway that regulates mitochondrial energy metabolism (Tort et al., 2014; Cronan, 2020). PDHA 1 (a key component of the pyruvate dehydrogenase complex) controls pyruvate entry into the TCA cycle (Echeverri et al., 2021). Univariate Cox’s regression confirmed that the FDX1, DLD, LIAS, LIPT1, and PDHA1 levels were all associated with OS, but only the FDX1 level independently predicted survival.

The soluble iron-sulfur protein ferredoxin 1 (FDX1) plays major roles in many metabolic pathways, for example, by transferring electrons during mitochondrial redox reactions (Sawyer and Winkler, 2017). Wang et al. (2021) found that FDX1 was involved in mitochondrial steroid metabolism, and played a role in the development of polycystic ovary syndrome. FDX1 facilitates copper-dependent cell death; an analysis of FDX1 action shed light on how cells respond to proteotoxic stress (Tsvetkov et al., 2019). FDX1 increases ATP production and plays key roles in glucose metabolism, fatty acid oxidation, and amino acid metabolism in lung adenocarcinoma cells (Zhang et al., 2021).

Any prognostic utility of glioma FDX1 status remains unclear. We found that FDX1 status is strongly correlated with glioma prognosis, the WHO glioma grade, IDH status, and 1p19q co-deletion status. The FDX1 level independently predicted glioma prognosis. Immune cells play important roles in tumor creation and development (Chasov et al., 2020). We explored the connections between FDX1 levels and immune cell populations. Higher FDX1 expression strongly enhanced tumor infiltration of macrophages, eosinophils, and Th2 cells but reduced infiltration of pDCs, NK CD56 bright cells, and Treg cells. Thus, FDX1 may regulate immune infiltration into the glioma microenvironment.

Our work had several limitations. Most data were mined from public databases and validated only *in vitro*; some of our findings require further validation. We interrogated only a few databases. Prospective studies with larger sample sizes are needed.

## 5 Conclusion

We explored the roles played by CRGs in glioma development. Our prognostic risk score based on the expression signature is strongly predictive of OS. The FDX1 level was independently prognostic; targeting of FDX1 expression might serve as a cuproptosis-specific therapeutic strategy for glioma prevention and treatment.

## Data Availability

The original contributions presented in the study are included in the article/Supplementary Material, further inquiries can be directed to the corresponding authors.

## References

[B1] Babak M. V. Ahn D. (2021). Modulation of intracellular copper levels as the mechanism of action of anticancer copper complexes: Clinical relevance. Biomedicines 9, 852. 10.3390/biomedicines9080852 34440056 PMC8389626

[B2] Blockhuys S. Celauro E. Hildesjo C. Feizi A. Stal O. Fierro-Gonzalez J. C. (2017). Defining the human copper proteome and analysis of its expression variation in cancers. Metallomics 9, 112–123. 10.1039/c6mt00202a 27942658

[B3] Brito C. Azevedo A. Esteves S. Marques A. R. Martins C. Costa I. (2019). Clinical insights gained by refining the 2016 who classification of diffuse gliomas with: Egfr amplification, tert mutations, pten deletion and mgmt methylation. Bmc Cancer 19, 968. 10.1186/s12885-019-6177-0 31623593 PMC6798410

[B4] Chasov V. Mirgayazova R. Zmievskaya E. Khadiullina R. Valiullina A. Stephenson C. J. (2020). Key players in the mutant p53 team: Small molecules, gene editing, immunotherapy. Front. Oncol. 10, 1460. 10.3389/fonc.2020.01460 32974171 PMC7461930

[B5] Chen B. Zhou X. Yang L. Zhou H. Meng M. Zhang L. (2022). A cuproptosis activation scoring model predicts neoplasm-immunity interactions and personalized treatments in glioma. Comput. Biol. Med. 148, 105924. 10.1016/j.compbiomed.2022.105924 35964468

[B6] Chen R. Smith-Cohn M. Cohen A. L. Colman H. (2017). Glioma subclassifications and their clinical significance. Neurotherapeutics 14, 284–297. 10.1007/s13311-017-0519-x 28281173 PMC5398991

[B7] Cronan J. E. (2020). Progress in the enzymology of the mitochondrial diseases of lipoic acid requiring enzymes. Front. Genet. 11, 510. 10.3389/fgene.2020.00510 32508887 PMC7253636

[B8] Dixon S. J. Lemberg K. M. Lamprecht M. R. Skouta R. Zaitsev E. M. Gleason C. E. (2012). Ferroptosis: An iron-dependent form of nonapoptotic cell death. Cell 149, 1060–1072. 10.1016/j.cell.2012.03.042 22632970 PMC3367386

[B9] Dorsam B. Fahrer J. (2016). The disulfide compound alpha-lipoic acid and its derivatives: A novel class of anticancer agents targeting mitochondria. Cancer Lett. 371, 12–19. 10.1016/j.canlet.2015.11.019 26604131

[B10] Duarte I. F. Caio J. Moedas M. F. Rodrigues L. A. Leandro A. P. Rivera I. A. (2021). Dihydrolipoamide dehydrogenase, pyruvate oxidation, and acetylation-dependent mechanisms intersecting drug iatrogenesis. Cell. Mol. Life Sci. 78, 7451–7468. 10.1007/s00018-021-03996-3 34718827 PMC11072406

[B11] Echeverri R. N. Mohan V. Wu J. Scott S. Kreamer M. Benej M. (2021). Dynamic regulation of mitochondrial pyruvate metabolism is necessary for orthotopic pancreatic tumor growth. Cancer Metab. 9, 39. 10.1186/s40170-021-00275-4 34749809 PMC8577026

[B12] Ge E. J. Bush A. I. Casini A. Cobine P. A. Cross J. R. DeNicola G. M. (2022). Connecting copper and cancer: From transition metal signalling to metalloplasia. Nat. Rev. Cancer 22, 102–113. 10.1038/s41568-021-00417-2 34764459 PMC8810673

[B13] Gilbert M. R. Dignam J. J. Armstrong T. S. Wefel J. S. Blumenthal D. T. Vogelbaum M. A. (2014). A randomized trial of bevacizumab for newly diagnosed glioblastoma. N. Engl. J. Med. 370, 699–708. 10.1056/NEJMoa1308573 24552317 PMC4201043

[B14] Gramatzki D. Dehler S. Rushing E. J. Zaugg K. Hofer S. Yonekawa Y. (2016). Glioblastoma in the canton of zurich, Switzerland revisited: 2005 to 2009. Cancer 122, 2206–2215. 10.1002/cncr.30023 27088883

[B15] Guo X. Wang T. Huang G. Li R. Da C. C. Li H. (2021). Rediscovering potential molecular targets for glioma therapy through the analysis of the cell of origin, microenvironment and metabolism. Curr. Cancer Drug Targets 21, 558–574. 10.2174/1568009621666210504091722 33949933

[B16] Hatori Y. Yan Y. Schmidt K. Furukawa E. Hasan N. M. Yang N. (2016). Neuronal differentiation is associated with a redox-regulated increase of copper flow to the secretory pathway. Nat. Commun. 7, 10640. 10.1038/ncomms10640 26879543 PMC4757759

[B17] Ishida S. Andreux P. Poitry-Yamate C. Auwerx J. Hanahan D. (2013). Bioavailable copper modulates oxidative phosphorylation and growth of tumors. Proc. Natl. Acad. Sci. U. S. A. 110, 19507–19512. 10.1073/pnas.1318431110 24218578 PMC3845132

[B18] Lah T. T. Novak M. Breznik B. (2020). Brain malignancies: Glioblastoma and brain metastases. Semin. Cancer Biol. 60, 262–273. 10.1016/j.semcancer.2019.10.010 31654711

[B19] Liang C. Zhang X. Yang M. Dong X. (2019). Recent progress in ferroptosis inducers for cancer therapy. Adv. Mat. 31, e1904197. 10.1002/adma.201904197 31595562

[B20] Muller B. J. Kulasinghe A. Chua B. Day B. W. Punyadeera C. (2020). Circulating biomarkers in patients with glioblastoma. Br. J. Cancer 122, 295–305. 10.1038/s41416-019-0603-6 31666668 PMC7000822

[B21] Ouyang Z. Zhang H. Lin W. Su J. Wang X. (2022). Bioinformatic profiling identifies the glutaminase to be a potential novel cuproptosis-related biomarker for glioma. Front. Cell Dev. Biol. 10, 982439. 10.3389/fcell.2022.982439 36158220 PMC9500213

[B22] Quinones A. Le A. (2018). The multifaceted metabolism of glioblastoma. Adv. Exp. Med. Biol. 1063, 59–72. 10.1007/978-3-319-77736-8_4 29946775

[B23] Reifenberger G. Wirsching H. G. Knobbe-Thomsen C. B. Weller M. (2017). Advances in the molecular genetics of gliomas - implications for classification and therapy. Nat. Rev. Clin. Oncol. 14, 434–452. 10.1038/nrclinonc.2016.204 28031556

[B24] Rouse C. Gittleman H. Ostrom Q. T. Kruchko C. Barnholtz-Sloan J. S. (2010). Years of potential life lost for brain and cns tumors relative to other cancers in adults in the United States. Neuro. Oncol. 18, 70–77. 10.1093/neuonc/nov249 PMC4677421 26459813

[B25] Sawyer A. Winkler M. (2017). Evolution of chlamydomonas reinhardtii ferredoxins and their interactions with [fefe]-hydrogenases. Photosynth. Res. 134, 307–316. 10.1007/s11120-017-0409-4 28620699

[B26] Tort F. Ferrer-Cortes X. Thio M. Navarro-Sastre A. Matalonga L. Quintana E. (2014). Mutations in the lipoyltransferase lipt1 gene cause a fatal disease associated with a specific lipoylation defect of the 2-ketoacid dehydrogenase complexes. Hum. Mol. Genet. 23, 1907–1915. 10.1093/hmg/ddt585 24256811

[B27] Tsvetkov P. Coy S. Petrova B. Dreishpoon M. Verma A. Abdusamad M. (2022). Copper induces cell death by targeting lipoylated tca cycle proteins. Science 375, 1254–1261. 10.1126/science.abf0529 35298263 PMC9273333

[B28] Tsvetkov P. Detappe A. Cai K. Keys H. R. Brune Z. Ying W. (2019). Author correction: Mitochondrial metabolism promotes adaptation to proteotoxic stress. Nat. Chem. Biol. 15, 757. 10.1038/s41589-019-0315-5 PMC8189136 31164776

[B29] Wang W. Lu Z. Wang M. Liu Z. Wu B. Yang C. (2022). The cuproptosis-related signature associated with the tumor environment and prognosis of patients with glioma. Front. Immunol. 13, 998236. 10.3389/fimmu.2022.998236 36110851 PMC9468372

[B30] Wang Y. Zhang L. Zhou F. (2022). Cuproptosis: A new form of programmed cell death. Cell. Mol. Immunol. 19, 867–868. 10.1038/s41423-022-00866-1 35459854 PMC9338229

[B31] Wang Z. Dong H. Yang L. Yi P. Wang Q. Huang D. (2021). The role of fdx1 in granulosa cell of polycystic ovary syndrome (pcos). BMC Endocr. Disord. 21, 119. 10.1186/s12902-021-00775-w 34130686 PMC8207664

[B32] Weger M. Weger B. D. Gorling B. Poschet G. Yildiz M. Hell R. (2018). Glucocorticoid deficiency causes transcriptional and post-transcriptional reprogramming of glutamine metabolism. Ebiomedicine 36, 376–389. 10.1016/j.ebiom.2018.09.024 30266295 PMC6197330

[B33] Yan X. Wang N. Dong J. Wang F. Zhang J. Hu X. (2022). A cuproptosis-related lncrnas signature for prognosis, chemotherapy, and immune checkpoint blockade therapy of low-grade glioma. Front. Mol. Biosci. 9, 966843. 10.3389/fmolb.2022.966843 36060266 PMC9428515

[B34] Ye Z. Zhang S. Cai J. Ye L. Gao L. Wang Y. (2022). Development and validation of cuproptosis-associated prognostic signatures in who 2/3 glioma. Front. Oncol. 12, 967159. 10.3389/fonc.2022.967159 36059638 PMC9434124

[B35] Zhang Z. Ma Y. Guo X. Du Y. Zhu Q. Wang X. (2021). Fdx1 can impact the prognosis and mediate the metabolism of lung adenocarcinoma. Front. Pharmacol. 12, 749134. 10.3389/fphar.2021.749134 34690780 PMC8531531

