# Supplementary material for: A novel prognostic signature of cuproptosis-related genes and the prognostic value of FDX1 in gliomas
Source: Front Genet. 2022 Dec 12;13:992995. doi: 10.3389/fgene.2022.992995 (PMC9792093; doi:10.3389/fgene.2022.992995)
Supplement: Supplementary file 4 [file DataSheet1.DOCX]

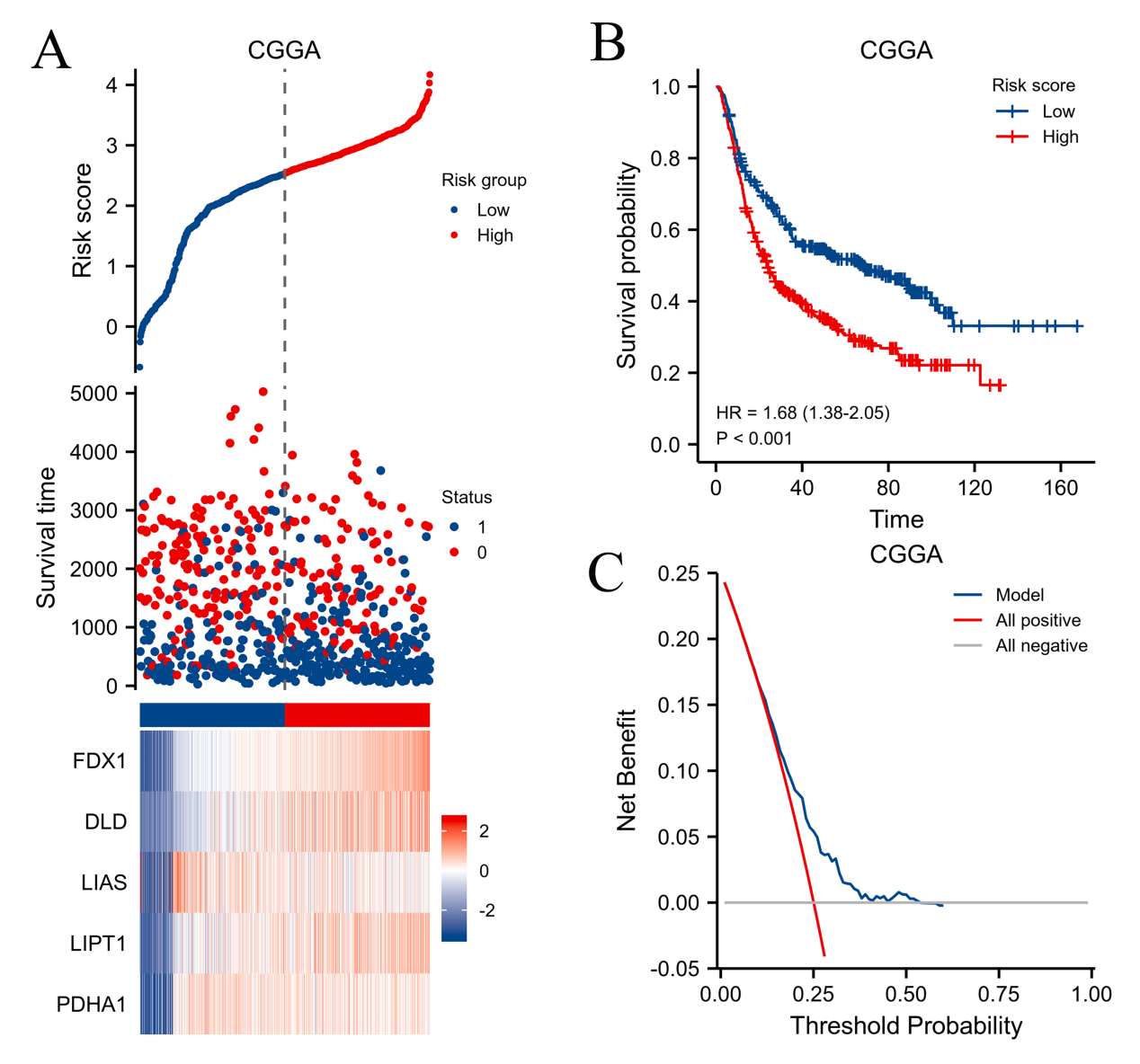


Figure S1. Clinical relevance of CRGs in the gliomas patients of CGGA. (A) Distribution of risk score, survival status and the expression of prognostic gliomas, (B) Kaplan−Meier plot of the CRGs signature and overall survival, (C) Decision curve analysis of CRGs signature for predicting survival status.
